# Facing the threat of influenza pandemic - roles of and implications to general practitioners

**DOI:** 10.1186/1471-2458-10-661

**Published:** 2010-11-02

**Authors:** Albert Lee, Antonio AT Chuh

**Affiliations:** 1 School of Public Health and Primary Care, The Chinese University of Hong Kong, 4th Floor, School of Public Health, Prince of Wales Hospital, Shatin, N.T., Hong Kong; 2 Adjunct Professor of Applied Health Science, School of Health, Physical Education and Recreation, Indiana University, Bloomington, Indiana, USA

## Abstract

The 2009 pandemic of H1N1 influenza, compounded with seasonal influenza, posed a global challenge. Despite the announcement of post-pandemic period on 10 August 2010 by theWHO, H1N1 (2009) virus would continue to circulate as a seasonal virus for some years and national health authorities should remain vigilant due to unpredictable behaviour of the virus. Majority of the world population is living in countries with inadequate resources to purchase vaccines and stockpile antiviral drugs. Basic hygienic measures such as wearing face masks and the hygienic practice of hand washing could reduce the spread of the respiratory viruses. However, the imminent issue is translating these measures into day-to-day practice. The experience from Severe Acute Respiratory Syndrome (SARS) in Hong Kong has shown that general practitioners (GPs) were willing to discharge their duties despite risks of getting infected themselves. SARS event has highlighted the inadequate interface between primary and secondary care and valuable health care resources were thus inappropriately matched to community needs.

There are various ways for GPs to contribute in combating the influenza pandemic. They are prompt in detecting and monitoring epidemics and mini-epidemics of viral illnesses in the community. They can empower and raise the health literacy of the community such as advocating personal hygiene and other precautious measures. GPs could also assist in the development of protocols for primary care management of patients with flu-like illnesses and conduct clinical audits on the standards of preventive and treatment measures. GPs with adequate liaison with public health agencies would facilitate early diagnosis of patients with influenza.

In this article, we summarise the primary care actions for phases 4-6 of the pandemic. We shall discuss the novel roles of GPs as alternative source of health care for patients who would otherwise be cared for in the secondary care level. The health care system would thus remain sustainable during the public health crisis.

## Background

The outbreak of Novel Influenza A (H1N1) has caused a global challenge since the first case was identified on 23 April 2009. Within nine weeks, all six WHO regions of the world were affected [1]. The impact of this pandemic is compounded by the ageing population in many countries and the new epidemics of "non-communicable diseases" [2]. More than 399,000 laboratory-confirmed cases from 192 countries were identified by 16 October 2009 [1]. Although WHO announced the post-pandemic period on 10 August 2010, H1N1 (2009) virus would continue to circulate as a seasonal virus for some years and national health authorities should remain vigilant during the immediate post pandemic period due to unpredictable behaviour of the virus (WHO Pandemic H1N1 2009 briefing note 23).

A semi-quantitative study in Australia reported that additional daily presentations to general practice surgeries would be 20-200 presentations per day [3]. One of us (AL) serving in public primary care setting had to re-organise some designated clinics in the catchment area to manage patients with influenza-like illnesses. However, such venture induced an increase in workload demand of other clinics for chronic illnesses, subsequently leading to double burden of diseases. Another author (AC) working in a private primary care setting in the community experienced around 50% increase in workload demand for influenza-like presentations. If a new outbreak occurs, increase in patient load will be inevitable. Should the presentations, risks of complications, and the infectivity of the new influenza pandemic be different, the situation could be much worse. How should we build up the spare capacity to prepare for and respond to the pandemic if it arises again?

## Discussion

### Our capability of responding to the pandemic

Most countries have contingency planning. The Royal College of General Practitioners in the UK, for example, has issued clear guidelines for the management and control of pandemic influenza [4]. However, the less developed countries are experiencing difficulties in putting these guidelines in operation owing to inadequate stockpiles of antiviral drugs to go beyond rapid containment in supporting the mitigation efforts [5]. For the South-East Asian countries, the hospital bed capacity and medical personnel might not have the capacity to care for sudden surges of large number of patients [6]. During the early phase of the pandemic in May 2009, concerns of the delay in launching of the UK National Flu Line were raised in an article published in the *British Medical Journal*. The Line acted as the main route for the public to get advice and access to antiviral treatment [7]. Widespread community transmission of an infectious disease could overwhelm our health care system globally. Close collaboration with functional components of public health such as home-based care and primary health care is therefore indispensable [1].

Absenteeism amongst health care workers could pose another threat to the health care system with the prolonged periods of a pandemic. A US-based survey found that nearly half of the health care workers might fail to report for duties during an influenza pandemic, particularly the technical and supporting staff [8]. Another study reported that 28% of German health care workers might remain absent from work in order to protect themselves [9]. Results from UK study on randomly selected healthcare workers suggest that absenteeism could be as high as 85% at any point during a pandemic [10]. It has been estimated that a General Practitioner (GP) might expect to see 50 new cases per week for an average list size, which would rise to 100 at the height of the pandemic if 30% of other GPs were sick [11]. However, these studies were done prior to the H1N1 pandemic, and the data analyses were based on an H5N1 situation which might not be valid to be extrapolated for the H1N1 pandemic [12,13].

An UK study on how GPs responded to an influenza pandemic revealed that at least one-quarter of the respondents would respond poorly to such a pandemic [14]. Non-urban GPs were less prepared to an influenza pandemic as compared to urban GPs and also less likely to be aware of pandemic preparedness plans.

### How would primary care physicians help?

An article authored by Jennings *et al *outlined the multi-strategic approach to pandemic preparedness which would be categorised as non-pharmaceutical (public health) and pharmaceutical measures [15]. The former is aimed to reduce the social impacts such as social distancing by prompt case isolation, household quarantine, and closure of school and workplaces. Responce of health services with increasing number of possible flu cases and the existing care of other patients, risk communication, data collection and surveillance, and basic respiratory hygiene practices are all important public health measures. The pharmaceutical measures included vaccination, anti-viral medications, stockpiling of vaccines and drugs and co-ordinated effort in distribution. This would involve pre-pandemic vaccination and treatment of cases for secondary prevention.

Although vaccination for H1N1 is now available, it does not entail overall willingness to accept. A study in Hong Kong amongst 2,255 health care workers showed that the overall willingness to accept pre-pandemic H5N1 vaccine was only 28.4% during a WHO influenza pandemic alert phase 3 [16]. No significant change in the level of willingness to accept vaccine was observed despite an escalation to alert phase 5 [16]. Public health measures would be more effective with close collaboration between public health authorities and GPs.

GPs in UK were generally praised on their dedication and efforts during the pandemic [17]. Most of the influenza cases were diagnosed clinically by GPs, not virologically in the laboratories [18]. The Royal College of General Practitioners closely liaised with health authorities and external agencies in the battle against H1N1 influenza [17]. Daily updates were sent to their College Members, and formal guidelines were in place specifically for GPs in England [19] and Scotland [20]. Moreover, GPs had access to a dedicated email address flu@rcgp.org.uk for enquiries and support.

### Fundamental preventive measures: GPs as health educators and reassurance of well subjects

Preventive interventions are more effective in primary care setting which are not related to any one disease or organ system [21]. Effective primary care integrates vertical care concerning the management of specific diseases from primary to tertiary care as well as horizontal care with emphasis on addressing the needs of individuals, families and the community [22]. This is particularly important for preparing and responding to a pandemic of influenza. Although routine long term implementation of some physical measures to interrupt or reduce the spread of respiratory viruses could be difficult, a systematic review showed that simple and inexpensive interventions could be effective in reducing the spread of respiratory viruses [23]. Good infection control (comprising policies and procedures to prevent or minimise the risk of transmission) is a well known cornerstone of disease management and should be the focus of general practice management of respiratory outbreaks [24]. GPs should be in excellent position to self-demonstrate as well as educating patients on the pertinence and efficacies of hygienic measures. The imminent issue now is how to translate these basic personal hygienic measures into day-to-day practice.

GPs possess unique skills to empower patients, and patients felt that they are better helped and more understood by GPs [22]. GPs are in excellent positions to translate national guidelines into public health educationand put the daily lives of patients into context. They could also help to improve the health literacy of the community on infection control.

GPs in Hong Kong played this role during SARS. The Hong Kong Medical Association established *Doctors' Network *amongst GPs in different districts to support the local communities [25]. If GPs could play the role of health educators on preventive controls and the reinforcement of personal hygiene and other precautious measures in the community and serve as reliable resource persons to share and disseminate information to the community, the well subjects would thus be reassured. Their important role of GPs providing psycho-social support to the community during health crises is unique.

### The management of patients with influenza and continuous care of other patients

GPs are prompt in detecting and reporting epidemics and mini-epidemics of viral illnesses [26,27]. The epidemiological data obtained in primary care represents the best proxy measurements of the day-by-day prevalence of ailments in the community.

GPs can also assist in the development of protocols for primary care management of patients with flu-like illnesses in accordance to national guidelines to avoid missing cases while at the same time preventing panics in the community. A study conducted in Hong Kong revealed that GPs were amongst the first group of doctors performing clinical audits in their practice in order to improve the structures, processes, and outcomes of their services [28]. GPs could play the frontier role in management of patients with influenza without complications to allow unexpectedly large numbers of ill patients to be managed in the community. GPs could support the continuity of health care provision by acting as alternate source of health care for those patients who would otherwise attend specialist outpatient clinics, accident and emergency departments [29], or be hospitalised. Moreover, GPs are trained as generalists, so they can manage a diverse range of health problems. In some countries fully trained GPs are capable of providing counselling to patients, their families, and alerted members in the community.

### Pharmaceutical measures for pandemic control - the role of GPs

Pharmaceutical measures for pandemic preparedness include the provision of vaccines particularly during the pre-pandemic period, and anti-viral drugs for treatment of cases as well as secondary prevention for selective cases. Although it was expected that the Australian Health Management Plan for Pandemic Influenza (AHMPPI) would enable frontline Australian GPs to maintain a central role during the Swine flu pandemic, their task was rendered extremely difficult owing to deficiencies in the implementation of AHMPPI [30]. This included resource supply failures, time-consuming administrative burdens, delays in receiving laboratory test results and approval for providing oseltamivir to patients, and a lack of clear communication about policy changes as the situation progressed [30].

Better consultation with front-line clinicians, particularly GPs, is crucial; and this must occur as a matter of high priority. Different countries have initiatives for GPs to play their roles during the flu pandemic [3,30]. Of specific interest is the "flu champion" in some Australian practices, which actively advocates educational activities, promotes vaccinations, and ascertains the availability of antiviral medications [24]. GPs in the UK can access influenza vaccines subject to their clinical discretion during serious periods of the pandemic [31]. No effort was spared to assure that GPs would continue their services during the pandemic [32].

### Collaborative role of GPs with Public Health Authorities: Learning from SARS experience

The outbreak of SARS in Hong Kong exposed the lack of support and guidance for GPs and other primary healthcare professionals during a public health crisis [33,34]. Despite fear, anxiety and uncertainty, GPs in Hong Kong demonstrated their willingness and commitments to discharge their duties as healers [34]. A study showed that 83.2% of GPs aspired deeper involvements in the war against SARS in the community - as educators (74.6%), as gatekeepers (68.4%), utilising rapid diagnostic tests (68.4%), and administering vaccines when available (84.2%) [34]. Some GPs expressed their wish to "share the government's outpatient burden and/or outreach services at elderly homes", and one doctor had volunteered to serve in a SARS screening clinic [34].

In terms of public health measures taken by GPs, more measures were taken by GPs in Hong Kong when compared to those in Toronto [35]. As the outbreaks were larger in scale and occurred at the community level in Hong Kong, the sense of vulnerability for possible infection in GPs should be higher in Hong Kong. However GPs in private practice voluntarily incurred negative commercial initiatives such as sharing patient loads, supplying appropriate protective barriers including expensive masks, delivering lectures in school and community centres, and being medical advisors for deprived members of the society such as inmates in elderly homes. Those initiatives would become public health actions with public health authorities drawing up the action plans for the GPs.

The SARS experience also revealed that patients were also unnecessarily referred to secondary care because of ineffective communications and the unavailability of some investigations to GPs. Valuable health care resources were thus inappropriately matched to community needs [36]. This would put even heavier burdens on the health care system during the outbreak of influenza.

If the public health authorities would work more closely with GPs with more rapid communications in clinical information, epidemiological update and results of investigations, GPs being the first point of contact for most patients in the health care system would provide better, comprehensive and continuing care during the public health crisis. Studies in Singapore, Australia and UK all showed the willingness of GPs to provide professional services during pandemic [10,37]. The findings were in sharp contrast to the sarcastic remarks by Dawes in an editorial "Caring for patient is a moral imperative during a pandemic influenza outbreak. I wouldn't be much of a human being if I closed up and headed for the hill."[11].

However the motivation was also altruistic as GPs participating in the Australian study did not have stockpiles of antiviral or personal protective equipments within their own practices [30]. They also believed that most appropriate setting to manage these patients was within GP practices and the government had a duty of care to stockpile on behalf of the GPs. Public health authorities would make good use of the public health initiatives currently in place in order to strengthen the roles of GPs within the system.

### How should GPs prepare for and respond to pandemics?

Reassurance of well subjects, the assessment and management of patients unwell with influenza, the continuous care of unaffected patients, and the attendance to the psychological consequences of the disaster were defined by an Australian study as key roles of GPs during pandemic [37]. GPs should provide optimal management for patients without flu-like illnesses, empower self care of patients, and act as alternate sources of health care for stable patients from secondary care. The ultimate goal is to enable GPs to relieve the workload of overwhelmed secondary care setting during the flu pandemic.

The framework for general practice by Nori and William described how to establish an effective level of infection control for different stages of outbreak [24]. Table 1 summarises the primary care actions for different components at the different pandemic phases (4-6) as defined by WHO [38].

**Table 1:** Roles of general practitioners in preparing for and responding to pandemics

	Phase 4	Phase 5 - 6
Running and Co-ordination	Co-ordination of triage system for suspected cases.	Liaison with national/local health authority for prioritisation of primary health care during pandemic.
	Standardisation of procedures in handling suspected cases and cautious cases.	Chair of GP network participates and gives advice in national/sub-national crisis committee.
	Action plan to avoid cross infection of suspected cases and other patients.	Co-ordination of care at primary care level for large influx of influenza patients and patients with other illness.
	Co-ordination of other sectors to care for large number of ill patients.	Provide local leadership in rational use of multi-sectoral resources in meeting the local health needs and demand.
	Identify the vulnerable and at risk groups for necessary health protection.	Care for those under medical surveillance.
	Co-ordination of care for close contacts and family members of suspected or cautious cases.	Protocol for home management for those with minor illnesses

Situation monitoring and Assessment	Collect specimens from suspected cases for rapid diagnosis.	Cases reporting
	Collect more clinical and epidemiological data from suspected cases.	Assess the capacity to manage larger number of ill patients.
	Monitoring of symptoms and signs of flu - like illness of close contacts.	Monitoring of caution cases.
	Close monitoring of 'suspected' or 'cautious' cases.	Assess the uptake and impact of mitigation measures at community level.
		Monitor resources in primary health care to meet the surge of demands as alternative health care for hospital setting in management of non-infectious diseases.

	Phase 4	Phase 5 - 6

Reducing the spread of disease	Clinical management of those fever cases or suspected cases according to national guidelines to avoid cross infection	Re-designation of clinics within the local catchment area to designated clinics for management of fever cases and those with flu-like illness, and other clinics to manage illness of non-infectious nature
	Working closely with local health authority for management of those suspected cases isolated at home and their households and close contacts	Home visits for patients with chronic illnesses rather than coming to clinics by those primary care doctors not involved in management of flu cases
	Health protection for at risk groups including clinic staff	Special review clinics for those requiring follow up for chronic illnesses to avoid cross infection
	Re-organisation of clinic schedule to minimise cross infection with minimal disruption of usual services	Prophylactic treatment for high risk groups
	To reinforce the community implementation of individual/household and societal level of disease control measures^34^	
	Given advice and provision of care to patients returning from high risk areas or close contact of suspected cases in accordance to national guidelines	

Continuity of Health Care and Provision	To activate the system in primary health care to manage flu-like illnesses as well as non-infectious illnesses with minimum chance of cross-infection	Primary care clinics as alternate source of medical care for those stabilised hospital patients with 'non-communicable diseases' to relieve the workload of hospital setting
	Self management protocol for patients with minor illnesses	Provide psycho-social support to patients, communities and also health care workers

Communication	Explain to local community what is known and not known about the virus, the state of outbreak, the effective preventive measures and next steps	Act as resource persons for community to have update information of transmission pattern, clinical severity, treatment and prophylaxis options
	Act as resource persons for enquire how to obtain medicines, essential services	Feedback of community concerns to national authority

The framework of Table 1 goes beyond infection control at clinic level. It also covers measures to handle the suspected cases and close contacts, advice to patients returning from high risk areas, and the identification of high risk cases. This framework also enables the primary care system to play a leading role to sustain the health care services during the pandemic so that the health care system can cope with a large influx of patients with influenza like illnesses without jeopardising the care of chronic illnesses patients.

Primary health care should be more proactive as an alternate sources of health care for hospital patients with stable conditions, developing of protocol for self management for certain illnesses, acting as resource persons for patient health education in the community and providing leadership to re-organise the local resources meeting the local health care needs.

It is highly crucial that such primary care action plan should be made readily available to GPs not only during but also before a pandemic. All levels of primary care professionals from administrators to individual GP surgery professionals and allied health professionals should be alerted to the existence and elements of these action plans. When resources including time and manpower are available, mini-drills could be conducted in surgeries to investigate the practicalities and logistical barriers of these actions. We also recommend conducting clinical audits to assess the structure, processes and outcomes of these primary care actions.

### What should be the next step?

The success of primary care in handling emerging health crises prompts us to re-conceptualise primary care as the foundation of care for all people rather than the mere provision of basic services for the lower strata of the society [21]. This is particularly important for developing countries where the delivery of primary care is usually more fragmented, rendering the entire health care system more vulnerable to the emergence of an influenza pandemic.

The roles of GPs should also be broadened to take up greater share of patient care in the entire health care system, particularly the co-ordination of triage systems for suspected cases, disease prevention and health promotion, improvement of health literacy of the community, alternate sources of care for patients in secondary care, surveillance, and close monitoring of suspected cases and/or close contacts. Clinical audits should be conducted to assess whether the actions are being implemented effectively and to identify barriers of implementing such actions in order to enact remedial solutions. We are convinced that implementation of the aforementioned measures would create a "win-win" situation for the community as well as all levels of the medical and healthcare systems on a global perspective.

## Competing interests

The author declares that they have no competing interests.

## Authors' contributions

AL is a general practitioner and an academic in the field of general practice and public health. His ideas of the paper come from researching, working experience with WHO as temporary advisor on many occasions, training of health professionals in disease prevention and health promotion, review of current evidence, and experience as general practitioner in different settings. AC is a general practitioner and an academic in general practice. His ideas of the paper come from researching, community services, training of health professionals, review of current literature, and experience as a general practitioner. Both are authors of the paper and contributed to initial idea, and to the serial drafts and agreed the final submission.

## Pre-publication history

The pre-publication history for this paper can be accessed here:

http://www.biomedcentral.com/1471-2458/10/661/prepub
